# Development of a three-item short form of the EAT-10 for screening suspected dysphagia in hospitalized older adults: findings from a multicentre study

**DOI:** 10.3389/fnut.2026.1927025

**Published:** 2026-09-03

**Authors:** María Trinidad Castillo-García, Irene Llagostera-Reverter, Joaquín Moncho-Vasallo, Víctor M. González-Chordá, Cristina Carretero-Randez, María Isabel Orts-Cortés, NUTRIFAG Group

**Affiliations:** 1 Dr. Balmis University General Hospital, Institute for Health and Biomedical Research (ISABIAL, Group 20), Alicante, Spain; 2 Department of Nursing, University of Alicante, Alicante, Spain; 3 Nursing Department, Nursing Research Group (GIENF Code 241), University Jaume I, Castellón de la Plana, Spain; 4 Research Unit for the Analysis of Mortality and Health Statistics, Department of Community Nursing, Preventive Medicine, Public Health and History of Science, University of Alicante, Alicante Institute for Health and Biomedical Research (ISABIAL), Alicante, Spain; 5 Nursing and Healthcare Research Unit (Investén-isciii), Instituto de Salud Carlos III, Madrid, Spain; 6 CIBER of Frailty and Healthy Aging (CIBERFES), Madrid, Spain; 7 Nursing Research Group: Infection, Inflammation and Chronicity (IIS La Fe), Hospital University and Polytechnic La Fe, Valencia, Spain; 8 Department of Nursing, University of Alicante (BALMIS), Dr. Balmis General University Hospital, Alicante Institute for Health and Biomedical Research (ISABIAL, Group 23), Alicante, Spain

**Keywords:** aged, deglutition disorders, dysphagia, mass screening, nursing assessment

## Abstract

**Aim:**

To develop a three-item short form of the EAT-10 by identifying the smallest subset of items that preserves the screening performance of the full questionnaire in hospitalized older adults.

**Design:**

Multicentre cross-sectional observational study.

**Methods:**

The study was conducted in nine public hospitals in Spain within the NUTRIFAG project. Hospitalized patients aged ≥65 years with a length of stay ≥48 h were included. Patients receiving enteral or parenteral nutrition at admission, those in terminal condition, or with a primary diagnosis of cancer were excluded. Suspected dysphagia was assessed using the Eating Assessment Tool (EAT-10), with a score ≥3 considered positive. Multivariate logistic regression models with forward item selection were used to identify the most informative symptoms indicators. The sample was randomly divided into a training subset (60%) and an internal assessment subset (40%). Model performance was evaluated using Nagelkerke’s pseudo *R*^2^, the Hosmer–Lemeshow test, sensitivity, specificity, and the area under the receiver operating characteristic curve (AUC).

**Results:**

The sample included 2,519 participants. Three symptom indicators emerged as the most informative for identifying patients with suspected dysphagia: requiring extra effort to swallow liquids, requiring extra effort to swallow solids, and coughing while eating. Together, these indicators showed high agreement with the classification obtained using the full EAT-10 in the training phase (*R*^2^ = 0.861; sensitivity = 0.863; specificity = 0.979; AUC = 0.982), which similar classification performance in the internal assessment subset (sensitivity = 0.881; specificity = 0.984).

**Conclusion:**

The proposed three-item subset represents a shortened version of the EAT-10 that preserved much of the screening performance of the full questionnaire in hospitalized older adults. Although it may facilitate dysphagia screening by reproducing the classification obtained with the full EAT-10 in routine nursing assessment, independent external validation against clinical reference standards is required before its diagnostic use can be recommended.

## 1 Introduction

Oropharyngeal dysphagia is defined as difficulty or inability to swallow solid or liquid food from the oral cavity to the stomach and it accompanies various underlying conditions. It is estimated that, in Europe, up to 80% of patients remain undiagnosed and do not receive any form of treatment for this condition (1–3).

In 2016, the European Union Geriatric Medicine Society (EUGMS) and the European Society for Swallowing Disorders (ESSD) published a consensus document recognizing oropharyngeal dysphagia as a geriatric syndrome (4). It represents a clinically relevant problem in older adults due to its impact on health, functional status, and quality of life, as well as its association with increased healthcare utilization and related costs (5).

Dysphagia may affect 30–40% of individuals aged over 65 years, and up to 44% of hospitalized older adults. Its prevalence is higher among patients with neurodegenerative diseases (up to 80%) or following stroke (approximately 40%). In addition, it is associated with factors such as advanced age, functional impairment, sarcopenia, frailty, polypharmacy, and comorbidity (2, 3, 6).

The most frequent complications include impairments in the efficiency and safety of swallowing, which may lead to malnutrition, dehydration, and aspiration pneumonia (7). Therefore, early identification of dysphagia is essential to prevent adverse outcomes.

The management of oropharyngeal dysphagia is heterogeneous and largely depends on the resources available in each healthcare setting. A key challenge is to improve its recognition as a clinically relevant condition in routine practice, particularly among hospitalized older adults with high clinical complexity (8).

In the hospital setting, swallowing disorders are of particular importance not only due to the availability of professionals and resources, but also because hospitalization is frequently associated with acute conditions, functional decline, frailty, and decompensation of chronic diseases, all of which increase the risk of dysphagia (9). Systematic assessment of these problems, and especially the identification of suspected dysphagia, is essential to prevent complications and optimize clinical outcomes (8).

Screening approaches enable the early identification of patients with suspected dysphagia and facilitate timely interventions (10). In this context, the Eating Assessment Tool (EAT-10) is a validated, symptom-based instrument that supports the identification of individuals with suspected dysphagia (11, 12). Beyond the use of specific screening tools, the early recognition of swallowing-related symptoms is particularly relevant in nursing practice, as it may facilitate timely interventions and prevent complications among hospitalized older adults (13).

However, nurses working in acute care settings routinely perform multiple assessments within a limited time frame. Consequently, identifying a small number of highly informative clinical indicators may help support dysphagia recognition while reducing assessment burden and facilitating integration into comprehensive geriatric assessment processes (14).

In this context, identifying key swallowing-related indicators that capture clinically relevant information may facilitate the early recognition of dysphagia risk in routine practice. Such indicators could support nursing assessment in hospitalized older adults and contribute to more efficient and focused comprehensive care.

Therefore, the aim of this study was to develop a three-item short form of the EAT-10 by identifying the smallest subset of questionnaire items that preserves the screening performance of the full EAT-10 in hospitalized older adults.

## 2 Methods

### 2.1 Study design and setting

A cross-sectional observational study was conducted within the framework of the multicentre NUTRIFAG project (Impact of hospitalization on nutritional status and dysphagia risk in individuals aged 65 years and over), which aims to assess the risk of malnutrition and nutritional status at admission in patients aged ≥65 years hospitalized in units of the Spanish National Health System (15).

The present study was designed to develop a three-item short form of the EAT-10 by identifying the smallest subset of questionnaire items that preserved the screening performance of the full instrument in hospitalized older adults. The study was reported with consideration of relevant items from the Standards for Reporting Diagnostic Accuracy Studies (STARD) guidelines (16) to ensure transparent reporting of the modelling and validation procedures. However, the present study should not be interpreted as a diagnostic accuracy study against an independent clinical reference standard.

### 2.2 Participants

The study population consisted of patients admitted to medical and surgical hospital units across nine public hospitals. Patients aged ≥65 years with a hospital stay longer than 48 h at the time of inclusion were eligible. All participants provided written informed consent prior to enrolment.

Patients receiving enteral and/or parenteral nutrition at admission, those in terminal condition, or with a primary diagnosis of cancer were excluded. Participants were recruited between October 31, 2022, and April 1, 2024, resulting in a total sample of 2,519 participants.

### 2.3 Variables and measurements

Sociodemographic variables (age and sex) and variables related to the care process were collected, including type of admission (medical, surgical), type of admission process (urgent, scheduled), and type of hospital unit (medical, surgical, mixed).

Functional status was assessed using the Barthel Index (17); risk of pressure injuries using the Braden Scale (18); cognitive status using the Pfeiffer questionnaire (19); comorbidity using the Charlson Comorbidity Index (20); and frailty using the FRAIL questionnaire (21). Nutritional status variables were also collected, including actual or estimated body weight based on reference formulas (22) and body mass index (BMI).

The Eating Assessment Tool (EAT-10) was used to assess suspected dysphagia (11, 23). The dependent variable was the presence of suspected dysphagia, defined as a score ≥3 on the questionnaire. The independent variables were the 10 swallowing-related indicators included in the EAT-10.

### 2.4 Data collection procedure

Participant recruitment and data collection were standardized through a general protocol and adapted to each hospital’s workflow. Data were collected by nurses and dietitians trained within the NUTRIFAG project.

Data were collected and recorded within 48 h of admission into the REDCap platform (Research Electronic Data Capture), hosted by the Biomedical Research Institute of Alicante (ISABIAL), a secure web-based system for research data management (24).

Eligibility verification, informed consent, and variable collection were performed by trained professionals. To ensure consistency across sites, all personnel received specific training in anthropometric measurement techniques and standardized data recording procedures.

A 1-month pilot study was conducted prior to the main study to assess the feasibility of the recruitment and data collection process and to standardize procedures across centers. The pilot study was used exclusively to refine study procedures before participant recruitment began.

Materials and procedures for measurements were standardized, and continuous communication was maintained through periodic meetings and platform-based alerts. The availability of electronic medical records in most hospitals allowed for partial automation of data entry, increasing efficiency and accuracy.

The multidisciplinary team had extensive clinical experience, strengthening the study’s reliability. Audiovisual training resources were also made available on the project’s website^1^ to ensure methodological consistency across sites.

### 2.5 Statistical analysis

All statistical analyses were performed using the final cleaned dataset, comprising 2,519 participants with complete responses and no missing data. Clinical and functional variables were used to characterize the sample and for bivariate analysis. First, a descriptive analysis of all study variables was performed according to their nature. Categorical variables were summarized using frequencies and percentages, while continuous variables were expressed as means and standard deviations. Subsequently, bivariate analyses were conducted after verifying the assumptions for parametric testing. Correlations between EAT-10 items and suspected dysphagia were assessed using Spearman’s correlation coefficient.

The statistical analysis focused on evaluating the informative capacity of the swallowing-related indicators included in the EAT-10 in relation to the dependent variable, defined as the presence of suspected dysphagia (EAT-10 ≥ 3). The modelling strategy was designed to identify the smallest subset of EAT-10 items that preserved the screening performance of the full questionnaire, with the aim of developing a shorter version of the EAT-10 for use in hospitalized older adults. Accordingly, the analyses focused on item reduction rather than on the development of an independent diagnostic prediction model. Only these swallowing-related indicators were included as independent variables, without incorporating additional clinical or sociodemographic variables into the multivariate models. Each EAT-10 item, scored on a 5-point Likert scale (0–4), was entered into the logistic regression models as a continuous predictor. Items were not dichotomized or modelled as categorical (dummy) or ordinal factors.

The identification of the most informative set of swallowing-related indicators associated with suspected dysphagia was performed in two phases. In the first phase, multivariate logistic regression models were fitted using the full sample, with suspected dysphagia (positive/negative) as the dependent variable. The swallowing-related indicators assessed by the EAT-10 were introduced progressively into the model using a forward selection approach. Prior to model fitting, correlations between the individual EAT-10 items and the EAT-10-based classification of suspected dysphagia were explored as a preliminary descriptive step to characterize the relative contribution of each item before multivariable modelling. Because the outcome was derived from the same instrument, these correlations were not intended to demonstrate independent clinical associations with dysphagia but to support the item-selection process. At each step, the indicator with the highest explanatory and discriminative capacity was selected. The forward selection procedure comprised three sequential selection steps. At the first step, each of the 10 EAT-10 items was evaluated as a candidate single-item model. After selection of the first item, each of the nine remaining items was evaluated for addition to the model. At the third step, each of the eight remaining items was evaluated for addition to the two-item model. Thus, 27 candidate models were evaluated across the three selection steps (10 + 9 + 8). This procedure represented a sequential forward-selection process rather than an exhaustive evaluation of all possible item combinations. Linearity in the logit for the three items included in the final model was formally assessed using the Box–Tidwell approach, applying the transformation to each item score plus one to accommodate zero values.

Because the objective of the present study was to identify the smallest subset of EAT-10 items capable of preserving the screening performance of the full questionnaire, rather than to develop the statistically optimal prediction model, the forward selection procedure was guided by predefined thresholds of explanatory and classification performance (25). Accordingly, the selection process was stopped when a Nagelkerke’s pseudo-*R*^2^, sensitivity, and specificity all exceeded 0.85, as these thresholds were considered consistent with the objective of obtaining a parsimonious short form while maintaining high agreement with the full EAT-10. Potential optimism in model estimation for the final three-item model in the full sample was assessed using 1,000 bootstrap resamples. The three selected items were held fixed across bootstrap resamples, with model coefficients re-estimated in each resample. Therefore, this procedure assessed optimism associated with model estimation but did not account for optimism arising from the preceding item-selection process. An optimism-corrected AUC was calculated accordingly. In the second phase, the stability and performance of the selected indicator set were internally assessed. The sample was randomly divided into a training subset (60%) and an internal assessment subset (40%). A multivariate logistic regression model was fitted in the training subset using suspected dysphagia (positive/negative) as the dependent variable and the selected items as independent variables. The resulting item set was then applied to the internal assessment subset to evaluate the stability of its classification performance. Model convergence was assessed during likelihood estimation, and the final logistic regression model, in which the EAT-10 items were entered as continuous predictors, converged successfully without convergence warnings.

Model performance was evaluated using Nagelkerke’s pseudo *R*^2^, the Hosmer–Lemeshow goodness-of-fit test, sensitivity, specificity, and the area under the receiver operating characteristic curve (AUC) as indicators of discriminative ability (25). 95% confidence intervals were calculated using Wilson intervals for sensitivity and specificity, bootstrap percentile intervals for Nagelkerke’s pseudo-*R*^2^, and intervals for the AUC. A *p*-value <0.05 was considered statistically significant. Statistical analyses were performed using SPSS version 29.

### 2.6 Ethical considerations

Written informed consent was obtained from all participants after providing detailed information about the study objectives, procedures, and participation. The NUTRIFAG project was approved by the Ethics Committees (CEIM) of all participating hospitals. The coordinating center’s approval reference was PI2020-127 (ISABIAL, protocol no. 200026).

The study adhered to the principles of the Declaration of Helsinki (beneficence, non-maleficence, autonomy, and justice) and complied with current regulations, including the European Union Regulation 2016/679 on the protection of personal data and the Spanish Organic Law 3/2018 on Data Protection and Digital Rights Guarantee.

## 3 Results

### 3.1 Sample characteristics

The mean age of the sample was 79.35 years (SD = 8.08), with a mean body weight of 68.35 kg (SD = 16.79) and a mean body mass index (BMI) of 24.97 (SD = 6.02). Women represented 51.3% of the sample (*n* = 1,291). Regarding admission characteristics, 92.5% (*n* = 2,328) were admitted through the emergency department, 95.2% (*n* = 2,397) were hospitalized for medical conditions, 84.6% (*n* = 2,130) were admitted to medical units, and 82.7% (*n* = 2,068) were polymedicated.

The mean Braden Scale score was 18.68 (SD = 3.74), the mean Barthel Index score was 64.67 (SD = 35.45), the mean Pfeiffer questionnaire score was 2.17 (SD = 3.33), the mean FRAIL score was 2.11 (SD = 1.30), and the mean Charlson Comorbidity Index score was 5.89 (SD = 2.04).

As shown in Table 1, suspected dysphagia was more frequent among patients admitted for medical conditions, hospitalized in medical units, admitted through emergency, and those with polypharmacy. In addition, patients who were older and presented higher risk of pressure injuries, greater functional dependence, cognitive impairment, frailty, comorbidity, lower body weight, and lower BMI were more likely to present suspected dysphagia. Statistically significant differences were observed for all these variables (*p* < 0.001), except for sex (*p* = 0.325) and type of admission (*p* = 0.169).

**Table 1:** Sample characteristics and bivariate analysis according to suspected dysphagia.

Variables	EAT-10				Variables	EAT-10			
		Positive	Negative				Positive	Negative	
	%(n)^1^	%(n)^1^	%(n)^1^	p^3^		m(ds)^2^	m(ds)^2^	m(ds)^2^	p^3^
Sex					Age	79.35 (8.08)	82.21(7.54)	78.80 (8.06)	0.000**
Female	51.3(1291)	16.9(208)	83.1(1020)	0.325*					
Male	48.7(1228)	15.5(200)	84.5(1091)		Braden	18.68 (3.74)	15.35 (4.15)	19.32 (3.29)	0.000**
Process type									
Medical	95.2(2397)	16.7(400)	83.3(1997)	0.003*	Barthel	64.67 (35.45)	34.35 (34.38)	70.53 (32.55)	0.000**
Surgical	4.8(121)	6.6(8)	93.4 (113)						
Unit type					Pfeiffer	2.17 (3.33)	1.63 (2.92)	5.02 (3.82)	0.000**
Medical	84.6(2130)	16.8(358)	83.2(1772)	0.025*					
Surgical	3.7(92)	6.5(6)	93.5(86)		Frail	2.11 (1.30)	2.78 (1.03)	1.98 (1.31)	0.000**
Mixed	11.8(297)	14.8(44)	85.2(253)						
Admission type					Charlson	5.89 (2.04)	6.62 (1.99)	5.75 (2.01)	0.000**
Scheduled	7.5(190)	12.6(24)	87.4(166)	0.169*					
Emergency	92.5(2328)	16.5(383)	83.5(1945)		Weight	68.35 (16.79)	61.17 (16.74)	69.73 (16.45)	0.000**
Polypharmacy									
Yes	82.7(2068)	17.4(359)	82.6(1709)	0.000*	BMI	24.97 (6.02)	22.20 (6.08)	25.50 (5.86)	0.000**
No	17.3(432)	10.4(45)	89.6(387)						

1, percentage (sample); 2, mean (standard deviation); 3, *p*-value; * chi-square; ** Mann–Whitney *U*-test.

A total of 16.2% (*n* = 408) of participants presented suspected dysphagia according to the EAT-10. The item “swallowing liquids requires extra effort” was the one most frequently reported as a severe problem (2.9%; n = 73). As shown in Table 2, when classifying participants according to the presence or absence of suspected dysphagia, the proportion of patients with suspected dysphagia reached 100% at higher item score categories.

**Table 2:** Description of suspected dysphagia.

EAT-10			
	Total	Negative	Positive
Item	%^1^(n^2^)	%^1^(n^2^)	%^1^(n^2^)
My swallowing problem has caused me to lose weight			
0 no problem	92.6(2333)	90.1(2101)	9.9(232)
1	3.2(80)	11.3(9)	88.8(71)
2	1.8(45)	2.2(1)	97.8(44)
3	1(24)	0	100(24)
4 It’s a serious problem	1.5(37)	0	100(37)
My swallowing difficulties interfere with eating outside the home			
0 no problem	92.8(2338)	90.2(2108)	9.8(230)
1	2.9(72)	4.2(3)	95.8(69)
2	1.5(38)	0	100(38)
3	1.2(30)	0	100(30)
4 It’s a serious problem	1.6(41)	0	100(41)
Swallowing liquids requires extra effort			
0 no problem	83.7(2105)	96.7(2035)	3.3(70)
1	6.5(163)	41.7(68)	58.3(95)
2	4(101)	7.9(8)	92.1(93)
3	3.1(77)	0	100(77)
4 It’s a serious problem	2.9(73)	0	100(73)
Swallowing solids requires extra effort			
0 no problem	83.7(2109)	95.5(2015)	4.5(94)
1	8.1(204)	36.8(75)	63.2(129)
2	4.9(123)	17.1(21)	82.9(102)
3	1.5(38)	0	100(38)
4 It’s a serious problem	1.8(45)	0	100(45)
Swallowing pills requires extra effort			
0 no problem	84.5(2129)	94.6(2013)	5.4(116)
1	8.9(223)	38.1(85)	61.9(138)
2	2.9(73)	17.8(13)	82.2(60)
3	1.8(45)	0	100(45)
4 It’s a serious problem	1.9(49)	0	100(49)
Swallowing is painful.			
0 no problem	95.8(2414)	87.2(2104)	12.8(310)
1	1.7(42)	14.3(6)	85.7(36)
2	0.8(21)	4.8(1)	95.2(20)
3	0.6(14)	0	100(14)
4 It’s a serious problem	1.1(28)	0	100(28)
The pleasure of eating is affected by my swallowing problem			
0 no problem	92.5(2330)	90.4(2106)	9.6(224)
1	3.2(80)	6.3(5)	93.8(75)
2	1.7(43)	0	100(43)
3	1(25)	0	100(25)
4 It’s a serious problem	1.6(41)	0	100(41)
When I swallow, the food sticks			
0 no problem	90.9(2291)	91.1(2087)	8.9(204)
1	4.6(115)	20(23)	80(92)
2	2.4(60)	1.7(1)	98.3(59)
3	0.9(22)	0	100(22)
4 It’s a serious problem	1.2(31)	0	100(31)
I cough when I eat			
0 no problem	86.1(2168)	93.9(2036)	6.1(132)
1	7.1(179)	40.2(72)	59.8(107)
2	3.6(90)	3.3(3)	96.7(87)
3	1.5(39)	0	100(39)
4 It’s a serious problem	1.7(43)	0	100(43)
Swallowing is stressful			
0 no problem	93.5(2356)	89.5(2109)	10.5(247)
1	2.6(65)	3.1(2)	96.9(63)
2	1.4(35)	0	100(35)
3	1(24)	0	100(24)
4 It’s a serious problem	1.5(39)	0	100(39)

1, percentage; 2, sample.

Valid *n* = 2,519; missing = 0.

### 3.2 Correlations between swallowing-related indicators and suspected dysphagia

Regarding suspected dysphagia, a low correlation was observed for the swallowing-related indicator “swallowing is painful” (*ρ* = 0.437), while moderate to strong correlations were found for the indicators “swallowing is stressful” (*ρ* = 0.589), “my swallowing problem has caused me to lose weight” (*ρ* = 0.602), “my swallowing problem interferes with eating out” (*ρ* = 0.620), “the pleasure of eating is affected” (*ρ* = 0.628), “food sticks in my throat” (*ρ* = 0.630), and “swallowing pills requires extra effort” (*ρ* = 0.694). The strongest correlations were observed for the indicators “I cough when I eat” (*ρ* = 0.695), “swallowing solids requires extra effort” (*ρ* = 0.734), and “swallowing liquids requires extra effort” (*ρ* = 0.803). As shown in Table 3, all correlations were statistically significant (*p* < 0.05).

**Table 3:** Swallowing-related indicators and suspected dysphagia.

Items of the EAT-10 questionnaire	*ρ*	*p*
My swallowing problem has caused me to lose weight	0.602	<0.001
My swallowing difficulties interfere with eating outside the home	0.620	<0.001
Swallowing liquids requires extra effort	0.803	<0.001
Swallowing solids requires extra effort	0.734	<0.001
Swallowing pills requires extra effort	0.694	<0.001
Swallowing is painful.	0.437	<0.002
The pleasure of eating is affected by my swallowing problem	0.628	<0.001
When I swallow, the food sticks	0.630	<0.001
I cough when I eat.	0.695	<0.001
Swallowing is stressful	0.589	<0.001

*ρ*, Spearman’s correlation coefficient; *p*, *p*-value. Correlations are uncorrected part-whole correlations, as the EAT-10 total score includes the corresponding item, and should not be interpreted as corrected item-total correlations.

### 3.3 Identification of the most informative swallowing-related indicators associated with suspected dysphagia

Table 4 presents the process used to identify the set of swallowing-related indicators with the highest explanatory and classification performance for suspected dysphagia, based on the stepwise inclusion of the indicators assessed by the EAT-10 according to the criteria described in the Methods section. The first indicator incorporated was “Swallowing liquids takes extra effort”, followed by “Swallowing solids takes extra effort”. The final indicator included was “I cough when I eat”, reaching a Nagelkerke pseudo-*R*^2^ of 0.861 (95% CI: 0.829–0.896), with a sensitivity of 0.863 (95% CI: 0.812–0.899) and a specificity of 0.979 (95% CI: 0.969–0.985), and showing good calibration (Hosmer–Lemeshow test: *χ*^2^ = 1.064; *p* = 0.302). The Box–Tidwell assessment provided no evidence of departure from linearity in the logit for any of the three selected items (all *p* > 0.05; joint likelihood-ratio test: *χ*^2^ (3) = 2.90, *p* = 0.407). In the full sample, bootstrap correction for optimism in model estimation showed minimal optimism (apparent AUC = 0.9870; mean optimism = 0.00028; optimism-corrected AUC = 0.9867).

**Table 4:** Fit of multivariable logistic regression models in subsample 1 (Hosmer–Lemeshow test).

				Hosmer-Lemeshow				
Model	-2log Likelihood	Sig	*R*^2^ Nagelkerke (CI 95%)^e^	*χ* ^2^	gl	Sig	Sensitivity (CI 95%)^e^	Specificity (CI 95%)^e^
1	514.186	<0.001	0.711^a^(66.3–74.8)	0.147^b^	1	0.702*	83.8^c^(79.9–87.1)	96.2^d^(95.3–96.9)
2	348.129	<0.001	0.815^a^(79.1–84.9)	1.527^b^	1	0.217*	85.4^c^(81.6–88.5)	97.5^d^(96.7–98.1)
3	269.145	<0.001	0.861^a^(82.9–89.6)	1.064^b^	1	0.302*	86.3^c^(81.2–89.9)	97.9^d^(97.2–98.4)

Variables in the model: Swallowing liquids requires extra effort.

Variables in the model: Swallowing liquids requires extra effort; swallowing solids requires extra effort.

Variables in the model: Swallowing liquids requires extra effort; swallowing solids requires extra effort; I cough when I eat.

a, Nagelkerke pseudo-*R*^2^; b, chi-square (*χ*^2^); c, sensitivity; d, specificity; *p*-value; e, confidence interval.

Other swallowing-related indicators assessed by the EAT-10, such as “Swallowing pills takes extra effort”, showed correlation values similar to some of the selected indicators; however, their inclusion did not improve the explanatory or classification performance of the model and were therefore not retained in the final set.

### 3.4 Internal assessment of the selected indicator set

**Figure 1:**
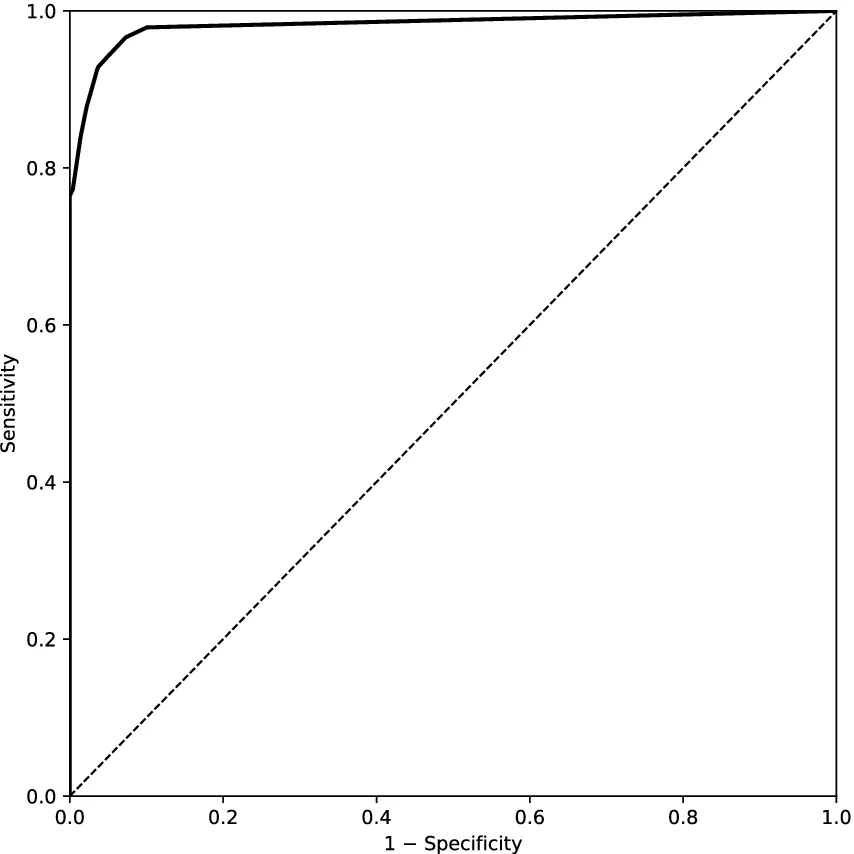
Receiver operating characteristic (ROC) curve of the final three-item EAT-10 subset in the training sample.

After identifying the final set of indicators in Phase I, Phase II focused on the internal assessment of their stability and classification performance. Table 5 shows the regression coefficients and adjusted odds ratios for each selected indicator, estimated using the training subsample. The adjusted odds ratios ranged from 11.864 to 15.937 (all *p* < 0.001), with magnitudes consistent with the order in which the items were entered into the model. Additionally, the ROC curve derived from the training subsample showed an area under the curve (AUC) of 0.982 (95% CI: 0.971–0.994), indicating that the proposed three-item subset closely reproduced the classification obtained using the full EAT-10. The corresponding ROC curve is shown in Figure 1.

**Table 5:** Final multivariable logistic regression model including three items, estimated using the training subsample.

				CI 95% OR^4^		
Item	B^1^	EE^2^	OR^3^	Lower	Upper	*p* ^5^
EAT-10 item 3. Swallowing liquids requires extra effort	2.769	0.270	15.937^1^	9.392	27.045	<0.001
EAT-10 item 4. Swallowing solids requires extra effort	2.536	0.242	12.626^1^	7.854	20.295	<0.001
EAT-10 item 9. I cough when I eat	2.473	0.320	11.864^1^	6.337	22.211	<0.001
Constant	−5.107	0.327				<0.001

1, B coefficient; 2, standard error; 3, odds ratio; 4, confidence interval; 5, *p*-value.

The selected indicator set was subsequently evaluated in internal assessment subset to assess the stability of its classification performance. The indicator set demonstrated high classification performance for suspected dysphagia, with a sensitivity of 0.881 (95% CI: 0.825–0.924) and a specificity of 0.984 (95% CI: 0.974–0.991), and an AUC of 0.995 (95% CI: 0.992–0.997).

## 4 Discussion

The aim of this study was to develop a three-item short form of the EAT-10 by identifying the smallest subset of questionnaire items that preserved the screening performance of the full instrument in hospitalized older adults. The findings suggest that three indicators “Swallowing liquids requires extra effort”, “Swallowing solids requires extra effort”, and “I cough when I eat” preserved much of the screening performance of the full EAT-10, explaining 86.1% of the observed variability. These indicators represent clinically recognizable manifestations of swallowing difficulties and encompass key aspects of swallowing efficiency and safety. Difficulties swallowing liquids and solids may reflect impairments in swallowing efficiency, whereas coughing during eating is commonly associated with compromised swallowing safety. Importantly, these findings should be interpreted as the development of a shortened version of the EAT-10 rather than as an independent diagnostic prediction model. The proposed three-item subset was designed to preserve the screening performance of the full questionnaire while potentially reducing respondent burden and facilitating its integration into routine clinical practice. However, these potential implementation benefits were not directly evaluated in the present study and should therefore be considered hypotheses for the future implementation research. From a nursing perspective, identifying a small number of highly informative swallowing-related indicators may be particularly relevant within the context of comprehensive nursing assessment tools. In this regard, instruments such as the VALENF Instrument (26, 27), aimed at the early detection of functional and safety-related problems in hospitalized patients, require brief and clinically meaningful indicators that can be easily integrated into routine practice. Within this framework, the proposed three-item short form could support the incorporation of suspected dysphagia assessment into a broader nursing assessment process, facilitating the early recognition of patients who may require further evaluation without the need to routinely administer the full EAT-10. This potential integration into the VALENF Instrument should be considered a future application requiring prospective evaluation rather than a validated implementation.

In relation to previous studies, the internal structure of the EAT-10 has been questioned using Rasch analysis (28). The present findings do not contradict those observations. Rather than addressing the measurement properties of the questionnaire, this study focused on developing a reduced version of the EAT-10 by identifying the most informative swallowing-related items for hospitalized older adults. The results suggest that a reduced subset of EAT-10 items preserved much of the screening performance of the full questionnaire, without implying improvements in the structural validity of the EAT-10.

Differences with other studies (6) may be explained by the characteristics of the study population. While that study focused on institutionalized patients with cognitive impairment, the present study includes a more heterogeneous sample of hospitalized older adults with diverse medical conditions and functional profiles, which may influence the relevance of specific swallowing-related indicators.

A prevalence of suspected dysphagia of 16.2% was identified, which is lower than that reported in previous studies (30–47%) (29, 30). This discrepancy is likely explained primarily by differences in the study populations and eligibility criteria, particularly the exclusion of patient groups with a high burden of dysphagia. Differences in the EAT-10 threshold used across studies (≥2 versus the original validated threshold of ≥3) may also have contributed to some variability in the reported prevalence. The present study followed the original validated EAT-10 cut-off (≥3), thereby maintaining consistency with the intended use of the instrument (11).

The EAT-10 remains a widely validated and accepted instrument for the identification of suspected dysphagia. Its diagnostic performance has been reinforced by meta-analyses, which recommend a threshold of ≥3 to optimize accuracy (31). The inclusion of objective confirmation tools, such as videofluoroscopy or fiberoptic endoscopic evaluation of swallowing (FEES), further supports its clinical value. Additionally, evidence highlights the relevance of screening even in patients without overt symptoms, as subclinical dysphagia may remain undetected (32). Therefore, the proposed three-item short form should not be interpreted as a replacement for the full EAT-10 or for established dysphagia screening and diagnostic procedures. Rather, it represents a shortened version of the EAT-10 that requires external validation against independent clinical reference standards before routine clinical implementation. Accordingly, the sensitivity, specificity, and area under the receiver operating characteristic curve (AUC) reported in this study should be interpreted as reflecting the agreement of the proposed three-item subset with the classification obtained using the full EAT-10, rather than its ability to detect true dysphagia. The high specificity observed should also be interpreted in light of the EAT-10-based outcome definition, as a score ≥3 on any single item is sufficient to classify a patient as having suspected dysphagia, introducing a structural component into the observed classification performance. Although the proposed three-item short form demonstrated high screening performance, no abbreviated screening instrument can completely eliminate the possibility of false-negative results. Accordingly, it should be used to support routine nursing assessment rather than as a substitute for comprehensive clinical evaluation. Patients with persistent clinical suspicion of dysphagia or other clinically relevant swallowing-related symptoms should undergo comprehensive swallowing assessment regardless of the screening result. Therefore, the proposed three-item short form should be considered a first-line screening aid to facilitate early identification and timely referral for further swallowing evaluation, rather than a replacement for clinical judgment or established dysphagia assessment procedures.

Furthermore, the factors associated with suspected dysphagia in this study, including advanced age, frailty, polypharmacy, functional dependence, and cognitive impairment, are consistent with previous literature and reinforce the importance of targeted screening strategies in high-risk populations (6).

Among the limitations, it should be noted that the dependent variable corresponded to suspected dysphagia as defined by the EAT-10 itself. Consequently, the proposed three-item short form should be interpreted as an item reduction exercise intended to preserve the screening performance of the full EAT-10 rather than as an independently validated diagnostic prediction model. Furthermore, the present study did not compare the proposed three-item subset with an external clinical reference standard, such as the Volume-Viscosity Swallow Test (V-VST), Fiberoptic Endoscopic Evaluation of Swallowing (FEES), or videofluoroscopic swallowing study (VFSS). Consequently, its diagnostic performance beyond the original EAT-10 remains to be established. Data collection for this external validation is currently underway and will be addressed in future research.

In addition, the study population excluded patients with a primary diagnosis of cancer, those receiving end-of-life care, and those requiring artificial nutrition. These eligibility criteria excluded patient groups known to have a high burden of dysphagia and therefore likely contributed to the lower prevalence of suspected dysphagia observed in our cohort compared with broader hospitalized older populations (33, 34). Consequently, caution is warranted when generalizing these findings to populations with a greater burden of swallowing disorders. Additionally, because indicator selection was performed using the full sample before the internal assessment of model performance, the reported performance should not be interpreted as an estimated obtained from an independent validation dataset and may overestimate the performance of the proposed three-item subset. Although bootstrap correction was applied to the final three-item model, this correction addressed optimism in model estimation with the selected items held fixed and did not account for optimism introduced by the item-selection process itself. In addition, the predefined stopping rule based on Nagelkerke’s pseudo-*R*^2^, sensitivity, and specificity >0.85 represents a non-standard model-selection criterion and should be considered when interpreting the selected three-item subset.

Future studies should perform item selection exclusively within the training sample and confirm these findings using independent external validation. Another limitation is that hospital-level clustering was not explicitly modelled. Future studies could evaluate the proposed three-item short form using hierarchical modelling approaches where appropriate. Nevertheless, the multicenter design and large sample size represent key strengths of the study, enhancing the representativeness of the findings.

## 5 Conclusion

The proposed three-item subset, comprising “Swallowing liquids requires extra effort,” “Swallowing solids requires extra effort,” and “I cough when I eat,” represents a shortened version of the EAT-10 that preserved the screening performance of the full questionnaire while reducing the number of items required for assessment. Its brevity may facilitate the incorporation of dysphagia screening into routine nursing assessment of hospitalized older adults, supporting the early identification of patients who may require further swallowing evaluation. However, independent external validation against objective clinical reference standards is required before its diagnostic use can be recommended.

## Data Availability

The datasets presented in this study can be found in online repositories. The names of the repository/repositories and accession number(s) can be found at: https://zenodo.org/records/14195477.

## Footnotes

1
https://nutrifag.isabial.es/
